# Top-down selection of visual working memory contents is supported by alpha-band phase-synchronized oscillatory networks

**DOI:** 10.1162/IMAG.a.1034

**Published:** 2025-12-23

**Authors:** Hamed Haque, Sheng H. Wang, Felix Siebenhühner, Edwin M. Robertson, J. Matias Palva, Satu Palva

**Affiliations:** Neuroscience Center, HiLIFE-Helsinki Institute of Life Science, University of Helsinki, Helsinki, Finland; BioMag Laboratory, HUS Medical Imaging Centre, Helsinki University Central Hospital, Helsinki, Finland; Department of Neuroscience and Biomedical Engineering (NBE), Aalto University, Espoo, Finland; Centre for Cognitive Neuroimaging (CCNi), School of Psychology and Neuroscience, University of Glasgow, Glasgow, United Kingdom

**Keywords:** MEG, oscillation, working memory, machine learning, synchronization

## Abstract

Visual working memory (VWM) maintenance depends on oscillatory network dynamics across multiple frequency bands throughout fronto-parietal and sensory brain areas. However, whether these networks reflect the active maintenance of visual information content or serve top-down control processes has remained unresolved. To address this, we used concurrent magneto- and electroencephalography (M/EEG) to measure brain activity during VWM tasks, in which the memory content was parametrically controlled. Using new edge-level analysis for source-connectivity networks, we disentangled connections and subnetworks underlying the maintenance of specific contents from those supporting feature-general VWM. We show here that long-range high-alpha band (α, 11–13 Hz) phase-synchronization networks carry out a dual role in these VWM functions. α-band subgraphs localized to the visual areas are feature-selective and maintain the contents of VWM. In contrast, the high α-band subgraph in the fronto-parietal areas was shared across memory contents, suggesting that it forms the content-agnostic executive core of VWM. We propose that α-band synchronization across distinct, but yet interconnected, subgraphs support the active maintenance of feature representations and their top-down selection.

## 1 Introduction

Visual Working Memory (VWM) maintains visual information online for use in cognitive operations or goal-directed behaviors. It has been suggested to comprise a distinct short-term storage of sensory information and executive processes (Baddeley, 2012; D’Esposito & Postle, 2015; Klingberg, 2010). In line with these models, functional magnetic resonance imaging (fMRI) studies have shown that WM consists of distributed activity across sensory cortices and fronto-parietal control networks (Emrich et al., 2013; Lee et al., 2013). While manipulation and executive control of VWM are consistently associated with prefrontal cortical (PFC) activity (Panichello & Buschman, 2021; Yu & Postle, 2021), it has remained under debate whether the representational functions arise within sensory cortices, the PFC, or from distributed activity (Christophel et al., 2017; Li & Curtis, 2023; Mejías & Wang, 2022; Yan et al., 2023).

In contrast to fMRI, electrophysiological methods yield direct mechanistic insight and have demonstrated that memory consolidation and refreshing are facilitated via neuronal oscillations (Benchenane et al., 2011). Oscillations are widely established to predict performance in VWM tasks in electroencephalography (EEG) (D. Wang et al., 2018; Pavlov & Kotchoubey, 2022; Ratcliffe et al., 2022; Sauseng et al., 2009), magnetoencephalography (MEG) (Honkanen et al., 2015; S. Palva et al., 2011; van Ede et al., 2017), and intracranial EEG (iEEG) (Bahramisharif et al., 2018; Johnson et al., 2023; ter Wal et al., 2021). These studies have further investigated the functional significance of oscillations in the maintenance of VWM contents vs. executive functions. VWM contents have been shown to be encoded by feature-selective spatio-temporal dynamics of beta (β, 20–30 Hz) and gamma (γ, 30–80 Hz) oscillations, as observed in MEG (Honkanen et al., 2015), intracranial EEG (Bahramisharif et al., 2018), and bursts in monkey PFC (Lundqvist et al., 2016, 2022). On the other hand, local alpha-band (α, 8–14 Hz) oscillations reflect distractor inhibition (Bonnefond & Jensen, 2012; de Vries et al., 2019; Magosso & Borra, 2024; Sghirripa et al., 2021; Zhou et al., 2023) and attention (Magosso et al., 2021; Riddle et al., 2020; Sattelberger et al., 2024; van Ede, 2018) of VWM.

In contrast to local oscillations, there is limited understanding of the functional significance of large-scale oscillatory networks in VWM. Scarce studies using EEG or MEG source connectivity analysis (Ericson et al., 2024; J. M. Palva et al., 2010; Magosso & Borra, 2024; Sato et al., 2018; Sattelberger et al., 2024) and local field potential (LFP) recordings in monkeys (Liebe et al., 2012; Salazar et al., 2012) have revealed that concurrent large-scale phase synchronization in multiple bands, that is, in α-, β-, and γ-bands, characterizes VWM performance. Recent work further suggests that inter-areal coupling in the form of traveling waves in the α-band could reflect top-down control in VWM (Zeng et al., 2024). However, it has remained unknown whether large-scale network oscillations would maintain VWM contents or mediate executive control over VWM processes, and further, in which frequency bands would these functions be mediated. One of the challenges is to identify parts of the complex brain network that are related to the given functions.

Here, we studied whether large-scale oscillatory networks would reflect VWM contents or their executive functions. Advances in brain network analysis (Markello et al., 2022; Shafiei et al., 2023), especially at the edge- (connection) level (Betzel et al., 2023; Faskowitz et al., 2022), provide a new way to chart neuronal architectures. We leveraged these approaches to identify subnetworks with VWM content-specific synchronization. We recorded ongoing brain activity with concurrent MEG and EEG (M/EEG) during a delayed match-to-sample VWM task in which we parametrically controlled VWM contents using a task paradigm in which the object statistical properties remained identical while only the to-be-remembered visual feature was varied. We then used edge-level data-driven source connectivity analysis to chart their distinct network architectures and content specific subgraphs This allowed us to resolve subgraphs encoding VWM contents and shared subnetwork across conditions, possibly reflecting executive control. Finally, we used machine learning (ML) for decoding the memory contents and established that phase-synchronization connectomes contain information of VWM contents.

## 2 Methods

### 2.1 Task and recordings

**Fig. 1.**
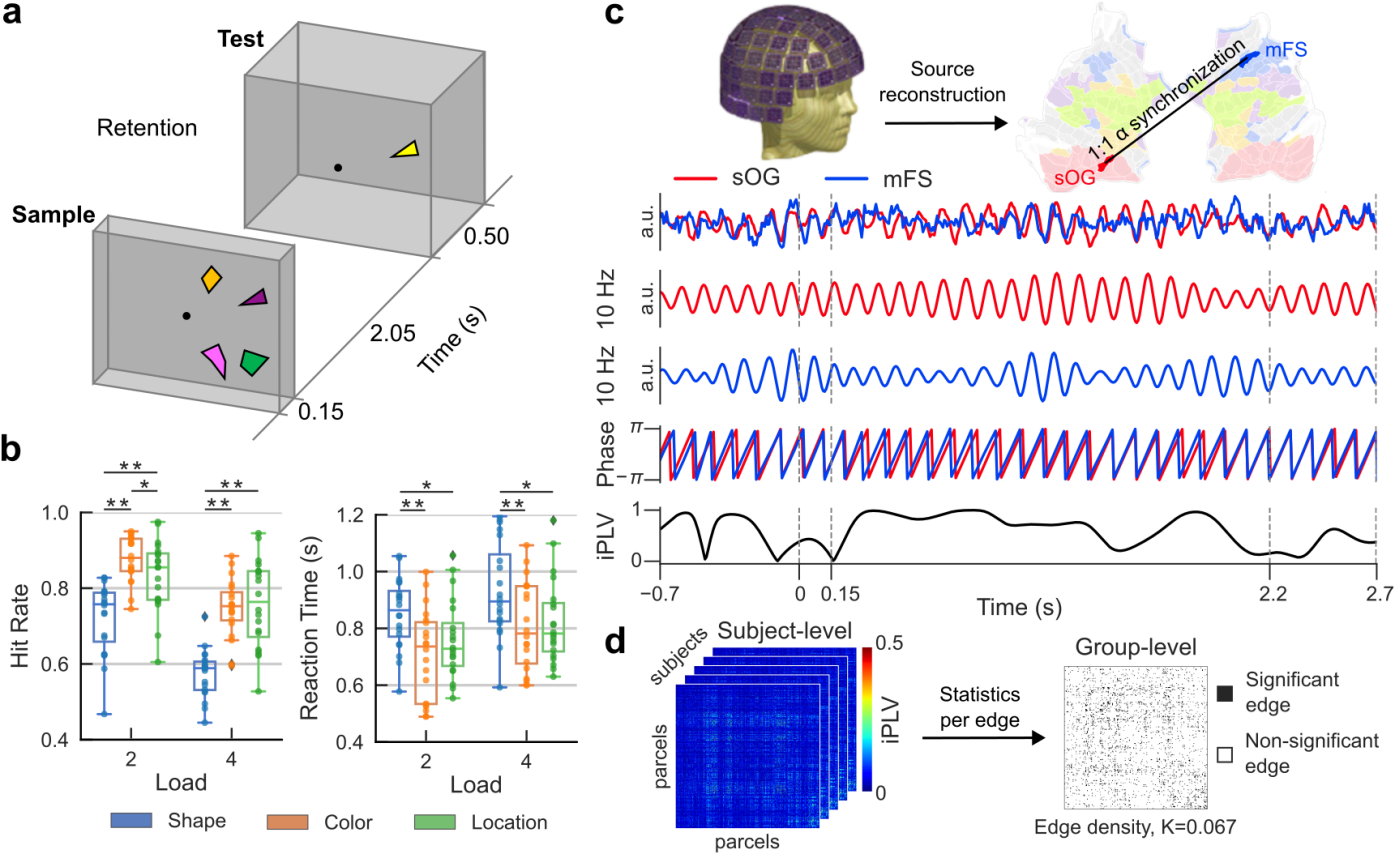
Schematic illustration of the experimental task and inter-areal synchronization during working memory retention*.* (a) Subjects memorized either the shapes, colors, or spatial locations for 2 loads (2 or 4 objects) in the Sample stimulus (S1, 0.15 s). S1 was followed by a 2.05 s retention period after which one object was presented as the Test stimulus (S2, 0.5 s) and the subject responded whether the memorized feature differed between S1 and S2 for this object. (b) Hit Rates (HRs) and Reaction Times (RTs) for different feature conditions (Shape, Color, and Location). Lines above boxplots represent significant differences (two-tailed repeated-measures t-test, *p < 0.01, **p < 0.001). (c) Data from M/EEG sensors were collapsed into cortical parcels. An example of prominent 1:1 phase synchronization of α-band oscillations in the left superior occipital gyrus (sOG, in red) and the right middle frontal sulcus (mFS, in blue) during VWM retention displayed in a flattened and inflated cortical surface. Red and blue traces show the Morlet filtered signals of sOG and mFS, respectively, with the signals’ phases below. The bottom panel shows time-resolved 1:1 synchronization between the two regions estimated with iPLV. (d) Synchronization matrices between all cortical parcels and for all Morlet-wavelet frequencies were computed for each subject. Statistical group-level analyses were performed at the edge level for each edge (connection) across subjects. This resulted a sparse weighted group-level statistical weighted adjacency matrix in which non-significant edges were set to zero, where the edge density, *K*, provides the fraction of significant connections.

The experimental procedure of the task and M/EEG recordings are described in detail in Honkanen et al. (2015). M/EEG data were collected from 20 healthy right-handed subjects (age 29 ± 6, mean ± SD, 8 females). The VWM task comprised three conditions in which subjects memorized the shapes, colors, or spatial locations of the objects in the “Sample” stimulus (S1, duration 150 ms) (Fig. 1a). After a 2050 ms retention period, a “Test” stimulus (S2, duration 500 ms) was presented and subjects indicated if it had the same or different task-relevant feature(s) as S1. The memorized stimuli were algorithmically generated for each trial and each stimulus was presented only once to avoid any long-term memory effects. For each subject, a total of 800 trials were recorded for each condition, to maximize within subject effect size (Ince et al., 2022).

Cortical activity was measured with concurrent M/EEG with 204 planar gradiometers, 102 magnetometers, and 60 EEG electrodes with a VectorView (Elekta Neuromag Ltd.) system at 600 Hz sampling rate at the BioMag Laboratory in Helsinki University Hospital. Ocular artifacts were measured with electro-oculogram (EOG) and the behavioral thumb-twitch responses with electromyography (EMG). T1-weighted anatomical MRI scans for cortical surface reconstruction models were obtained at a resolution of 1 x 1 x 1 mm using an MP-RAGE protocol with a 1.5 T MRI scanner (Siemens, Germany). The study was approved by the ethical committee of Helsinki University Central Hospital and was performed according to the Declaration of Helsinki. Written informed consent was obtained from each subject prior to the experiment.

### 2.2 Analysis of behavioral data

For each feature and memory load, we computed Hit Rates (HR) and Reaction Times (RTs) Performance differences between features and loads were also evaluated with both frequentist and Bayesian statistics. Repeated-measures t-test was used to compare the HRs and RTs across features and loads. Bayes factors were computed using the R package BayesFactor and provided an estimate of evidence for performance differences. We reported Bayes factors BF01 (Rouder et al., 2009) expressing the probability of the data given H0 (absence of an effect) relative to H1 (presence of an effect). BF01 values larger than 1 are therefore in favor of H0. For the Bayes factor analysis, an uninformative Jeffreys prior was placed on the variance of the normal population, while a Cauchy prior with scale parameter of r = √2/2 was placed on the standardized effect size.

### 2.3 Preprocessing of M/EEG data

An overview of the workflow including all analysis steps of the M/EEG data is shown in Supplementary Figure S1. We applied the temporal extension of signal space separation (tSSS) method with MaxFilter software (Elekta Neuromag) to the raw signal in MEG sensors to suppress extra-cranial noise, interpolate bad channels, and co-localize recordings in signal space individually for each subject. Independent component analysis (ICA, MATLAB toolbox Fieldtrip) was used to identify and extract components correlated with ocular (identified using the EOG signal) or heart-beat artefacts (identified using the magnetometer signal as a reference). The pre-processed M/EEG sensor time-series were epoched into individual trials of 2700 ms, spanning from −700 ms pre-S1 onset to 1800 ms post-S1. Time series data were then filtered into narrow-band time series using a bank of 38 complex Morlet wavelets with the time–frequency compromise parameter *m* = 5 and approximately log-linearly spaced center frequencies ranging from 3 to 120 Hz. After filtering, the narrow-band data were downsampled to a sampling rate of five times the center frequency.

### 2.4 Source modelling and cortical parcellation

To obtain accurate synchronization estimates, we used source-localized M/EEG data. FreeSurfer software (http://surfer.nmr.mgh.harvard.edu/) was used for volumetric segmentation of the MRI data, surface reconstruction, flattening, cortical parcellation, and neuroanatomical labeling with Destrieux atlas. MNE software (https://mne.tools/stable/index.html) was used for source modeling with minimum norm estimate using the dSPM method. The 148 parcels of the Destrieux atlas were split into 400 cortical parcels by iteratively splitting the largest parcels along a previously determined axis. The fine-grained parcellation was used to yield maximum sensitivity to individual differences in functional cortical anatomy as well as for robust optimization of the source-collapsing approach (Korhonen et al., 2014; Siebenhühner et al., 2016). Noise covariance matrices (NCMs) were obtained using preprocessed broad-band filtered M/EEG time-series (151–299 Hz) from 0.7 s time-windows prior to S1 onset. The NCMs were used to create MNE inverse operators to project the sensor-space M/EEG data into source space. The source models had dipole orientations fixed to the pial surface normals and a 5 mm inter-dipole separation. To reconstruct ongoing cortical phase dynamics, source narrowband complex vertex time series were collapsed into parcel time series in 400-parcels of the Destrieux-atlas using a source-reconstruction-accuracy (fidelity) weighted collapse operator (Korhonen et al., 2014). This enhanced the identification of true edges among spurious connections (S. Palva & Palva, 2012; Siebenhühner et al., 2016). Parcels were also assigned to one of the seven functional systems of the Yeo parcellation (Thomas Yeo et al., 2011) using a consensus mapping approach, where each parcel was assigned to the subsystem that contained the largest fraction of its vertices.

### 2.5 Analysis of oscillations amplitudes and inter-areal synchronization

Inter-areal phase-synchronization was computed in three separate time windows: 700 ms to 100 ms before S1 (baseline window), 600 ms to 1200 ms after S1 (early retention), and 1200 to 1800 ms after S1 (late retention). To allow maximal individual functional cortical separability and robust optimization of the source-collapsing approach, phase synchronization was estimated between all 400 parcels. We computed phase-synchronization for each wavelet frequency using the imaginary part of the complex phase-locking value (iPLV) (Lachaux et al., 1999; Rouhinen et al., 2020) that is insensitive to zero-lag interactions and hence yields neither artificial nor true zero lag couplings (J. M. Palva et al., 2018; S. H. Wang et al., 2018). For each subject, the inter-areal synchronization was computed by pooling the iPLV values across the given time-window and across trials of each condition (Shape, Color, Location). To combine the early and late retention periods, for each subject and condition, the iPLV values of each edge were averaged over the two retention-period time-windows. Oscillation amplitudes were computed for the same wavelet frequencies, parcels, and time-windows as the synchronization analysis, and pooled across all trials of the conditions (Shape, Color, Location) separately for each subject.

To obtain single-trial iPLV values and oscillation amplitudes for the classification analysis, for each subject and wavelet frequency, inter-areal synchronization and oscillation amplitudes were computed as described above, but the estimates were not averaged across trials for each condition. iPLV values were computed within each trial (and not across multiple trials) by estimating the instantaneous phase difference between parcel pairs across time points and then taking the imaginary part of the mean complex phase difference.

### 2.6 Statistical analyses

Before statistical testing, both oscillation amplitudes and synchronization were baseline-corrected by subtracting the mean amplitude or iPLV value of the pre-S1 baseline window for each wavelet frequency. The 400-parcel data was collapsed to a coarser 200-parcel parcellation to improve statistical stability by reducing the effects of inter-subject variability in functional anatomy (Rouhinen et al., 2020). Group statistical analyses were then carried out separately for each frequency, parcel (or parcel pair, for synchronization) and time window.

To identify significant retention period synchronization and its load dependence at the group level, we compared, for each wavelet frequency and each edge, the strength of synchronization averaged across loads against the strength at baseline, and also compared the strengths between individual loads, using the Wilcoxon Signed Rank test at α = 0.05. To estimate effect sizes for the identified significant differences between conditions, we estimated the average strength over edges for each participant. A repeated-measures *t*-test was then used to compare the strength of average synchronization between the conditions. We identified significant correlations between the strength of synchronization and individual HRs at the edge-level separately for each condition using a Pearson correlation test.

**Fig. 2.**
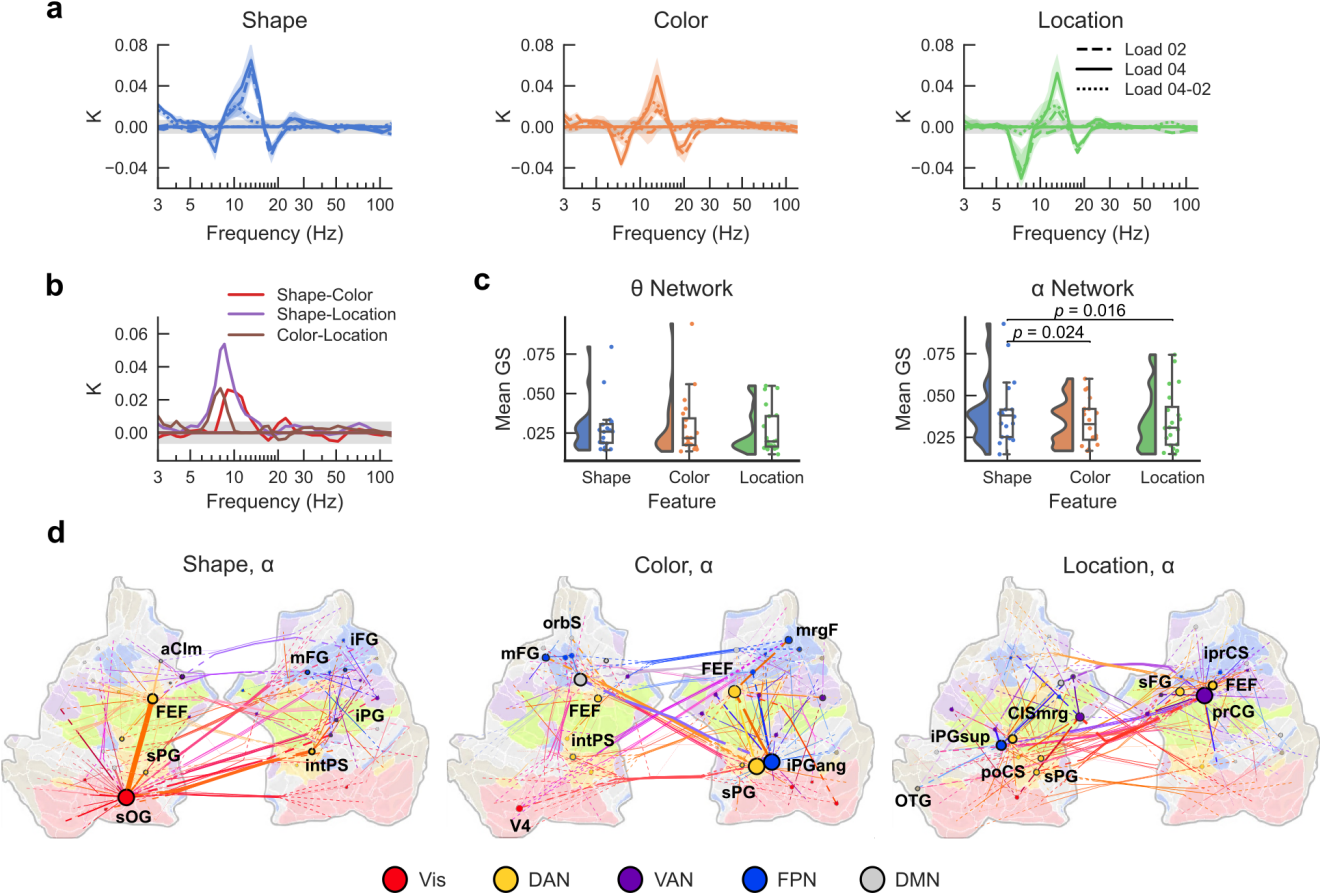
Content dependent large-scale synchronization and desynchronization characterizes VWM maintenance*.* (a) Edge density, *K,* as a function of frequency for the retention period for each condition (Shape, Color, Location), averaged across the Early and Late retention time windows, separately for each load compared to baseline as well as for the difference between the loads. Two-tailed Wilcoxon signed-rank tests were used to test if iPLV values of each edge were different from zero (*p* < 0.05). (b) Edge density (*K*) for the significant differences in synchronization strength between the conditions. (c) Distribution of individual graph strength (GS) values for the α (11–13 Hz) and θ (6–8 Hz) networks for the three features, edges selected based on (b). Two-tailed repeated-measures t-test was performed between each pair of features. (d) Graphs of α-band synchronization for each condition shown in **a,** displayed on an inflated and flattened cortical surface. The 200 strongest edges, as identified by edge betweenness centrality, and their connected nodes are shown. Connections are bundled into hyperedges (see section 2). Surface and node rendering reflect subsystem identity, with node size being proportional to node degree. In each hyperedge bundle, edge colors reflect the mixing of the colors of connected nodes, with the color being defined by Yeo subsystems. Yeo subsystems: visual network (Vis); dorsal attention network (DAN); ventral attention network (VAN); fronto-parietal network (FPN); default mode network (DMN).

To remove false positives (FP) caused by multiple statistical tests, we pooled all significant observations over all edges and then discarded as many of the least-significant comparisons as would be predicted to be false discoveries by the α level (J. M. Palva et al., 2010; Siebenhühner et al., 2020). To remove any remaining FPs, a threshold Q was defined for the number of significant observations that could arise by chance in any of the frequencies even after controlling for multiple comparisons (Puoliväli et al., 2020). This was estimated by simulating random processes at the null hypothesis, using the same number of tests performed in the actual experiment, and recording the residual fractions of “significant” observations after the elimination of the number of significant observations predicted to be false positives by the α level. Given the number of cortical parcels and time windows in which statistical analyses were performed for each condition, Q was estimated to correspond to 0.672% edge density and is shown as the shaded gray area in Figures 2 and 4.

### 2.7 Graph analysis and visualization

Spectro-temporal patterns of synchronization and amplitude modulations were visualized as time-frequency representations (TFRs). For each time-frequency (TF) bin, we computed the fraction of cortical parcels that showed a significant increase (*i.e.*, P+, the fraction of significant positive parcels for each TF bin) or decrease (i.e., P-, the fraction of significant negative parcels for each TF bin). For oscillation amplitudes, we localized the TF region of interest.

We used a graph theory (Bullmore & Sporns, 2009) approach to characterize the network structures as weighted graphs. Adjacency matrices were thresholded by statistical significance such that the non-significant edge values were set to zero. Adjacency matrices were then defined as graphs where the nodes were the cortical parcels and edges the significant interactions between nodes.

Edge density (*K*) was used to index the proportion of significant edges of all possible interactions and was computed as the number of significant edges divided by the total number of edges in the graph. Positive and negative values of *K* were used to indicate the proportion of edges showing a significant increase or decrease in synchronization strength, respectively.

In addition, graph strength (GS) was used to describe the mean strength of synchronization and was computed separately for each frequency and participant as the average strength over all significant connections.

To characterize inter-areal synchronization within and between functional subsystems (Yeo parcellation and functional visual subdivisions), for each subsystem pair, we computed edge density for all possible edges for the given pair. Within subsystems, edge density was computed for all edges between the parcels within the system. The differences in edge strengths between functional visual subdivisions were obtained by computing the difference between the average edge strengths (Δ-iPLV).

Node centrality metrics were further used to identify highly connected nodes in the graphs that putatively played a key role in network communication. Node degree was used to index how each node was connected to the other nodes, with a higher degree indicating more connections. Edge betweenness centrality, a measure of the number of shortest paths between node pairs that pass through an edge, was used as a measure to reveal the central core of the network for communication.

To remove residual signal leakage among nearby parcels (S. Palva & Palva, 2012) from the graphs, we excluded poorly source-reconstructed parcel edges from the graph analysis and visualizations. While using iPLV to estimate inter-areal synchronization would exclude the direct effects of zero-phase lagged signal mixing, spurious interactions would still remain (J. M. Palva et al., 2018). We therefore removed edges between parcels for which the source reconstruction accuracy (fidelity) was below 0.165 (6.5% of parcels). To further exclude spurious connections, we removed edges between parcels that exhibited greatest signal leakage with their neighbors, measured with cross-patch PLV (fidelity radius greater than 0.35). A total of 16.2% of all possible edges were thus excluded from the analyses. To further mitigate the contribution of spurious interactions caused by the concurrent presence of true interactions and linear mixing, edges were bundled by their adjacency signal mixing to hyper-edges (S. H. Wang et al., 2018). The hyper-edges thus bundled together edges that putatively originated from a single true edge among the spurious edges caused by source-leakage (S. Palva & Palva, 2012; J. M. Palva et al., 2018). These hyper-edges were used to visualize inter-areal network synchronization (Fig. 2).

### 2.8 Identifying shared and feature-specific edges

To identify the shared and feature-specific subnetworks, we created a union matrix by combining the retention-period matrices for the three visual features. The union matrix was created by combining the raw graphs of Shape, Color, and Location, using the edge or average edge strength across conditions. Next, we computed hyperedge bundling across the union matrix. Small hyperedges were excluded as they were likely to comprise false positives. To determine if a hyperedge was shared, a participation threshold was set whereby each of the three features (conditions) contributed a minimum proportion of raw edges to the hyperedge. To ensure that at least two conditions contributed substantially to the shared hyperedge, the participation threshold was set to > 0.3, normalized by the edge densities of the raw graphs. If the hyperedge did not meet this requirement for at least two conditions, it was considered condition-specific to the condition which contained the largest proportion of raw edges in the hyperedge.

### 2.9 Classification analysis of trials of single features

Machine learning (ML) was used to classify visual features, based on the retention period synchronization matrices and parcel-level oscillation amplitude patterns as features for the classifier using the random forest classification method. For each frequency and load, we first averaged iPLV values across all subjects and trials for the retention period subtracted by baseline data. The largest 1000 iPLV values of the group-averaged matrix were then used to create a binary mask that was applied to all trials of individual subjects. Classification on local oscillation amplitudes was performed using amplitude values from all 400 parcels for each trial.

Classification was performed at the level of individual subjects and separately for each narrow-band frequency. For each subject and Morlet wavelet frequency, single-trial iPLV matrices and oscillation amplitudes for the baseline and the two retention period windows (Early and Late) were computed, using the same frequency-specific filtering and synchronization estimation as in the group-level analyses. After applying the binary mask, this yielded a 1 x 1000 vector per trial for synchronization and a 1 x 400 vector per trial for oscillation amplitudes. A random forest classifier was then trained on these vectors of each trial using a leave-one-out cross-validation (LOOCV) method applied at the trial level (i.e., train on all trials minus one and then classify whether the left-out trial is from the Shape, Color, or Location condition, and then repeated for all trials). For each subject, and separately for each time window and load condition, 688 ± 75 (mean ± SD) trials were used in each classification run. Therefore, in each classification run (i.e., one LOOCV iteration), the classifier was trained on ~99.8% of trials and tested on the remaining ~0.2% (i.e., one trial). Classification accuracy was obtained as the proportion of trials correctly classified for each subject and averaged across the subjects. As participants memorized different visual features in separate experimental blocks, baseline activity already contained information about the relevant feature. Therefore, differences in the classification accuracy in the retention vs. baseline time-windows were obtained. We tested, for each frequency, whether the classification accuracy of the retention period significantly differed from the classification accuracy of the baseline period at the group level (Wilcoxon signed-rank test).

Classification was also applied to narrow-band amplitudes of each trial. The procedure was the same as for inter-areal synchronization, except the classification to decode the memorized feature was performed on the average parcel amplitudes.

## 3 Results

### 3.1 Behavioral performance

We used here a delayed match-to-sample VWM task for which we had earlier revealed that local γ-band amplitudes underlie the maintenance of feature-specific information in VWM (Honkanen et al., 2015) (Fig. 1a). We manipulated both VWM content and load such that the task was to memorize either the shapes, colors, or spatial locations of either 2 or 4 objects. Within each condition, the Hit Rate (HR) decreased with increasing memory load (two-tailed repeated measures t-test, *p* < 0.05) (Fig. 1b). Differences in HR between conditions were observed in both load conditions. HR for Shape was significantly lower than for Color (load 2: *t* = -8.018, *p* = 1.62e-07, BF01 = 8.62e-06; load 4: *t* = -1.62, *p* = 1.46e-12, BF01 = 1.53e-10; repeated measures t-test) and Location (load 2: *t* = -5.15, *p* = 5.66e-05, BF01 = 0.002; load 4: *t* = -8.72, *p* = 4.58e-08, BF01 = 2.64e-06) while HR for Color was significantly greater than Location only in load 2 (*t* = 2.88, *p* = 9.69e-03, BF01 = 0.21). Reaction Time (RT) increased with increasing memory load for each condition (two-tailed repeated-measures t-test, *p* < 0.05) (Fig. 1b). RT for Shape was significantly higher than for both Color (load 2: *t* = 5.69, *p* = 1.75e-05, BF01 = 0.00068; load 4: *t* = 4.00, *p* = 7.79e-04, BF01 = 0.022; repeated-measures t-test) and Location (load 2: *t* = 3.82, *p* = 1.16e-03, BF01 = 0.032; load 4: *t* = 3.43, *p* = 2.81e-03, BF01 = 0.072). These results indicate that, for both load 2 and 4, the task in the Shape condition was significantly more demanding than in both the Color and Location conditions.

### 3.2 Large-scale network synchronization differentiate memory contents

Networks of inter-areal phase-synchronization were computed from source-reconstructed MEG data in a data-driven manner (Supplementary Fig. S1). We estimated phase-synchronization using iPLV between all cortical parcels separately for each frequency and for the three time-windows (baseline and two retention period time windows) (Fig. 1c). Statistically significant network level synchronization were then characterized using graph theory (Bullmore & Sporns, 2009) such that cortical parcels (brain areas) were the nodes and statistically significant connections of synchronization were the edges of the network, the edge weight defining the strength of synchronization (see section 2). To characterize the extent of synchronization, for each time- and frequency-bin, we computed edge density *(K*) separately for statistically significant positive and negative interactions. Edge density was defined as the fraction of statistically significant inter-areal interactions of all possible pairwise interactions among the 400 brain areas for each condition (Fig. 1d).

We first averaged iPLV values over the three conditions (Shape, Color, Location) and over the two memory loads. This showed that the VWM retention period was characterized by dynamic sustained synchronization in the high-α-band (11–13 Hz) and concurrent desynchronization in the θ- (6–8 Hz) and β-bands (17–20 Hz compared to baseline (Supplementary Fig. S2, two-tailed Wilcoxon signed-rank test, *p* < 0.05). Having established that the VWM retention period is characterized by robust synchronization, we next computed phase synchronization for each condition (Shape, Color, and Location) compared to baseline separately for the two loads as well as for the difference between the loads. All features showed similar spectral patterns with robust α-band synchronization and concurrent suppression of θ- and β-band synchronization (two-tailed Wilcoxon signed-rank test, *p* < 0.05) (Fig. 2a). These patterns were more prominent in the late retention period (1.2–1.8 s) than in the early retention period (0.6–1.2 s) (Supplementary Fig. S3). As the load conditions showed similar synchronization patterns, we averaged data over the memory loads for the subsequent analyses. To compare with previous literature, we also computed local oscillation amplitudes for each feature and load condition. We found that VWM retention was characterized by a sustained and wide-spread decrease in the low-frequency (3–12 Hz) and β-band (15–25 Hz) oscillations amplitudes (Supplementary Fig. S4). As found previously (Honkanen et al., 2015), oscillation amplitudes were also load-dependently increased.

We next examined if synchronization would differ between the different conditions. The edge densities of both α-band synchronization and θ-band desynchronization differed between feature conditions (two-tailed Wilcoxon signed-rank test, *p* < 0.05) (Fig. 2b), demonstrating that both increased inter-areal synchronization and decreased (desynchronization) inter-areal synchronization reflect memorization of VWM contents. To define the effect sizes for the statistical analysis at the individual subject-level, we first defined the frequency bands in a data-driven manner using hierarchical clustering to reveal clusters of adjacent frequencies with spatial similarity. This revealed α-band (11–13 Hz) and θ-band (6–8 Hz) frequency clusters (Supplementary Fig. S5) that were used for the subsequent analysis. The graph strength (GS), which is the averaged strength over all significant connections across the whole brain, was then computed at the individual level and statistical significance between conditions estimated across participants. The α-band GS differed between feature conditions (two-tailed repeated-measures t-test, *p* < 0.05) (Fig. 2c), while the differences for θ-band desynchronization did not reach statistical significance. For the α-band synchronization, there was a significant difference in *GS* between the Shape and Color conditions (*t* = 2.45, *p* = 0.024), and between Shape and Location conditions (*t* = 2.66, *p* = 0.016). As the Shape condition was more difficult than Color and Location conditions, to ensure that results did not reflect differences in task difficulty, we compared the synchronization dynamics between conditions with similar HRs, that is, load 2 for Shape with load 4 for Color and Location conditions. We found condition-related differences in both α-band synchronization and θ-band desynchronization (Supplementary Fig. S6), indicating that both of these dynamic modulations reflect memory contents.

To then understand whether synchronization networks would connect task relevant cortical areas, we mapped the anatomical structure of the synchronization networks shown in Figure 2a. To this end, we plotted the edges and nodes with the highest edge-betweenness centrality, that is a centrality measure that quantifies an edge’s importance in a network by measuring the number of shortest paths between node pairs that pass through that edge and identifies edges that act as “bridges” connecting different parts of the network. To aid in the functional interpretation of the connections, only the edges within and between relevant cortical systems of visual, DAN, VAN, FPN, and default mode network (DMN) were visualized in Figure 2d with the complete networks shown in Supplementary Figure S7. Importantly, the functional network anatomy differed between conditions. For Shape, the most central hub was the left superior occipital gyrus (sOG), which connected to the key nodes of the dorsal attention network such as frontal eye fields (FEF, located in the superior precentral sulcus), intraparietal sulcus (intPS), and bilaterally to the visual cortex. The left sOG was also connected to the inferior frontal gyrus (iFG) and middle frontal gyrus (mFG) of the prefrontal cortical (PFC), and inferior parietal gyrus (iPG) of posterior parietal cortex (PPC). For Color, the most central hubs were the right superior parietal gyrus (sPG) and right inferior parietal gyrus (iPG) of the PPC, which were connected primarily to the left middle frontal gyrus (mFG). Synchronization was also strong across the attention networks, namely the dorsal attention network (DAN), ventral attention network (VAN), and the fronto-parietal network (FPN). For Location, the central hub was the right precentral gyrus (prCG) with bilateral connections to sPG and iPG. Importantly, FEF was a key node in all networks. The most central parcels for each of the networks are provided in Supplementary Table S1, along with the information of functional subsystems. The overall synchronization patterns thus demonstrated that α-band synchronization networks had central hubs in attentional control networks that were connected to functionally specialized visual areas.

### 3.3 α-band synchronization is localized to feature selective visual areas

**Fig. 3.**
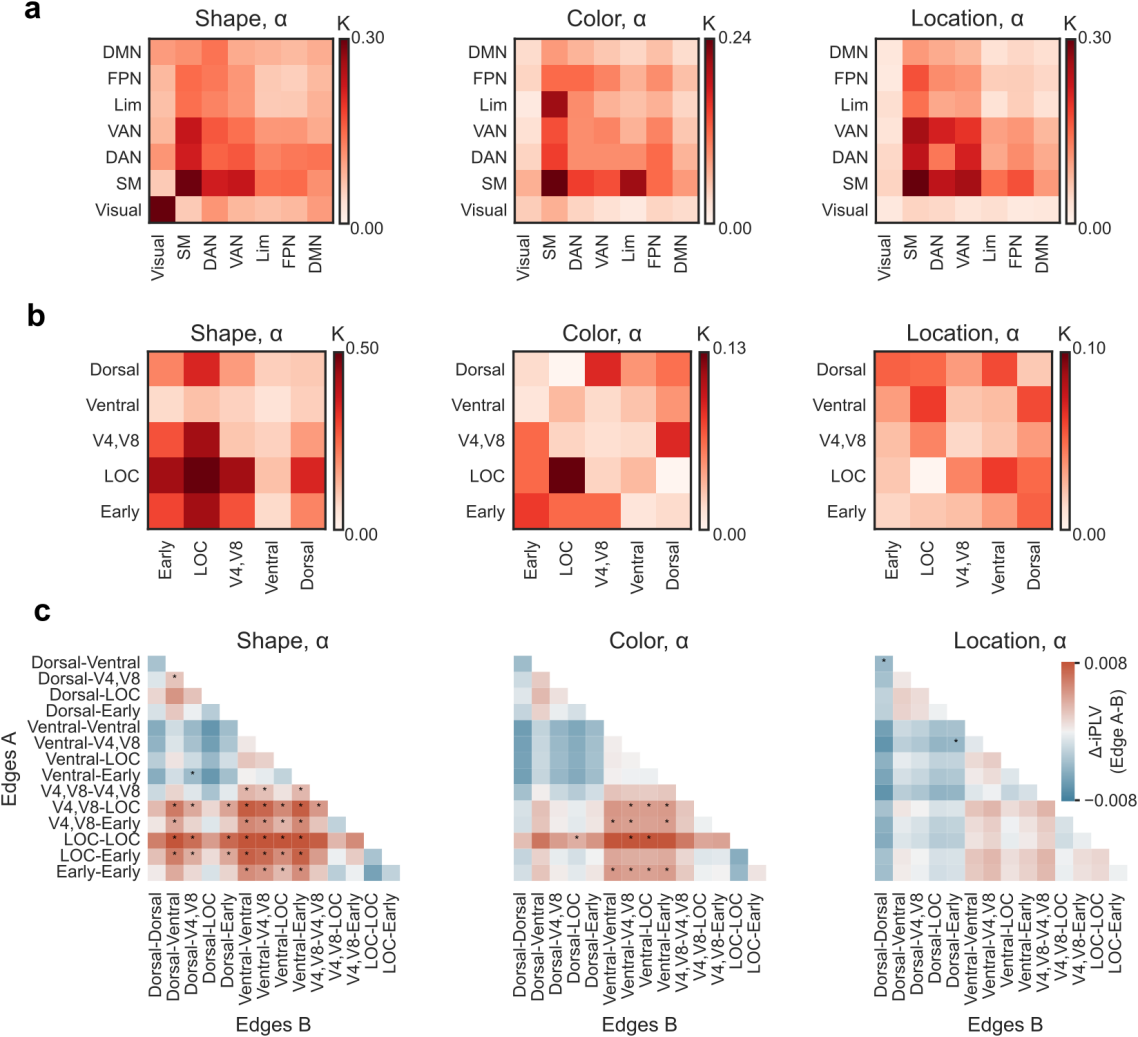
Functional localization of α-band synchronization. (a) Edge density for the α-band synchronization networks during the retention period for each condition (Shape, Color, Location) separately for within and between functional subsystems of the Yeo atlas. Yeo subsystems: visual network (Visual); somatomotor network (SM); dorsal attention network (DAN); ventral attention network (VAN); limbic network (Lim); fronto-parietal network (FPN); default mode network (DMN). (b) Edge density for the α-band synchronization networks within and between functional visual subdivisions. Visual subdivisions: early visual cortex, V1-V3 (Early); lateral occipital cortex (LOC); visual areas V4 and V8 (V4,V8); ventral stream visual areas (Ventral); dorsal stream visual areas (Dorsal). (c) The differences in mean phase synchronization (Δ-iPLV) between functional visual subdivisions (Edge A > Edge B) for the α-band synchronization networks in (b). The red color indicates stronger synchronization for edges in Edges A (y-axis) and blue for edges in Edges B (x-axis). Stars denote the subsystem pairs where the group difference was significant (two-tailed Wilcoxon-signed-rank test, *p < 0.05).

As the increase in α-band synchronization was feature-selective, we focused the subsequent analysis on this frequency band. To investigate the cortical network organization of the α-band synchronization across the functional networks, we co-localized the 400 parcels of the Destrieux atlas with fMRI-based functional systems of the Yeo atlas (Thomas Yeo et al., 2011) comprising of the visual network (Visual), somatomotor network (SM), dorsal attention network (DAN), ventral attention network (VAN), limbic network (Lim), fronto-parietal network (FPN), and the default mode network (DMN).We then estimated the strength of synchronization within and between the subsystems for each condition for data averaged across memory loads. For Shape, synchronization was robust within the visual system, as well as between and within the DAN, VAN, and SM; for Color in SM and its connections to Limbic system; and for Location within and between SM, VAN and DAN (Fig. 3a).

To further test if α-band synchronization would be specifically localized to visual areas responsible for processing of the memorized or distracting visual features, we assigned the parcels from the visual system to distinct functional visual subdivisions (Riesenhuber & Poggio, 2000, 2002): early visual cortex (V1-V3), lateral occipital cortex (LOC), ventral visual stream, and dorsal visual stream as in Honkanen et al. (2015) (Supplementary Fig. S8). We then estimated the number of significant edges within and across these functional visual systems (Fig. 3b).

The α-band synchronization for the Shape condition had the most connections in the early visual areas (V1-V3) and the lateral occipital cortex (LOC), that is, in areas that are involved in object perception (Hansen et al., 2007; Kravitz et al., 2013; Tootell & Hadjikhani, 2001), but also in the dorsal visual stream regions, whose activity is related to the processing of location information, although more recently it has also been connected to the (related) processing of shapes (Freud et al., 2017; Theys et al., 2015). For the Color condition, the strongest connections were found across the early visual areas as well as V4 and V8 and areas associated with color processing; for Location in the dorsal visual stream and in areas responsible for spatial information processing.

We then addressed differences in the mean edge strength between functional visual subdivisions for each condition (two-tailed Wilcoxon signed-rank test, (*p* < 0.05) (Fig. 3c)). For the Shape condition, we found that connections involving LOC, V4, and V8 showed significantly stronger synchronization than others. For Color, connection within and involving LOC and early visual areas dominated, while for Location, connections within and involving the dorsal stream were strongest. These data indicate that α-band synchronization is largest within and between the feature-relevant visual regions.

Albeit not in our main focus, to understand the functional role θ-band desynchronization may play in the VWM retention, we also plotted the anatomical structure of θ-band desynchronization. Intriguingly, desynchronization was found in the fronto-parietal attention networks connected with visual system areas (Supplementary Fig. S9). The θ-band desynchronization was widespread and not focused on visual areas, although it was consistently stronger for Shape and Color in LOC. Hence, θ-band desynchronization overlapped with that of strengthened α-band synchronization.

### 3.4 The fronto-parietal synchronization network transcends memory contents

**Fig. 4.**
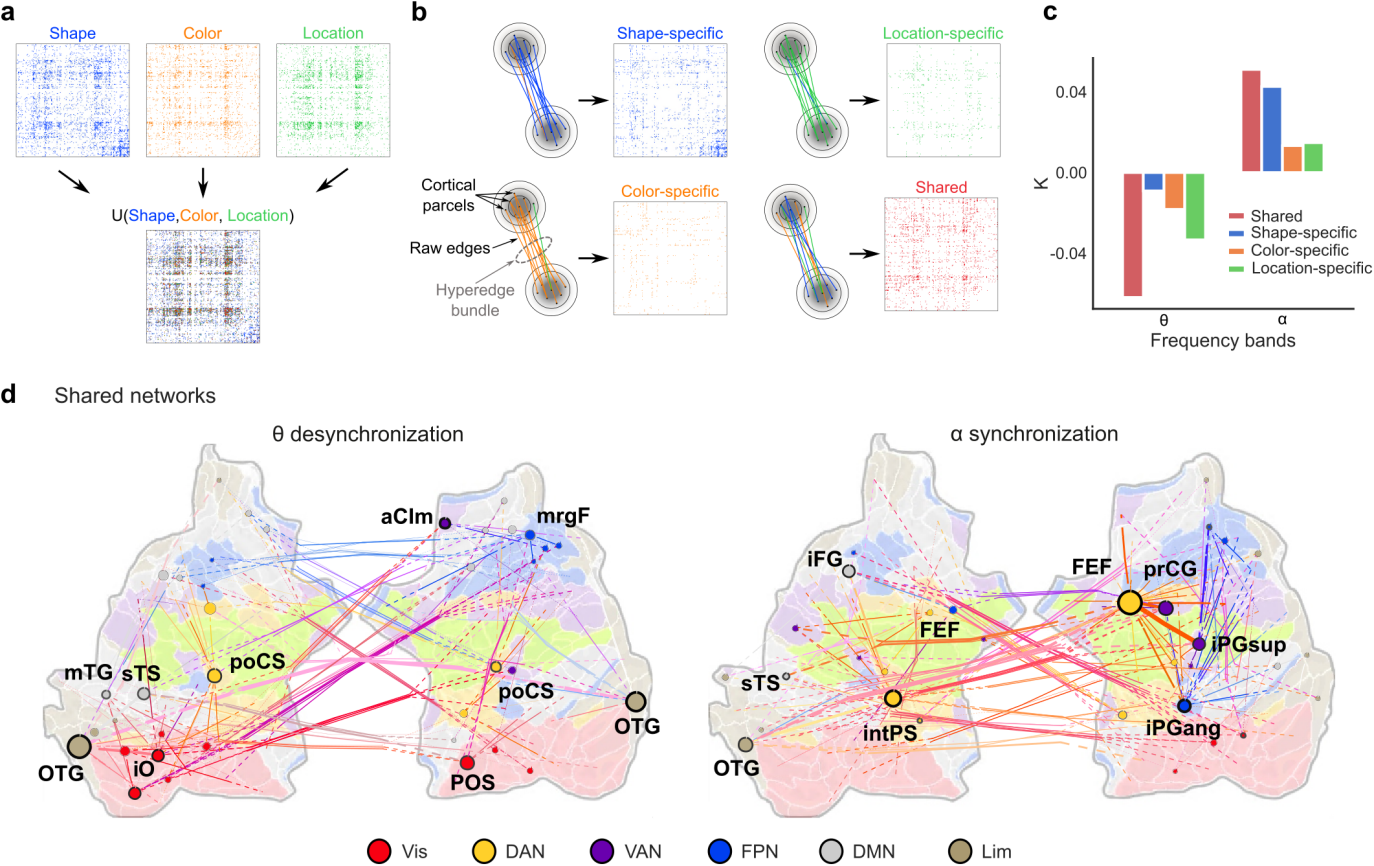
Shared networks connect visual and fronto-parietal regions*.* (a) Schematics of the approach. To identify feature-specific and shared networks, the group-level synchronization matrices of significant edges were combined into a union matrix. (b) All edges in the union matrix that putatively originated from the same single true edge, among the spurious edges caused by source-spread, were bundled together to a hyper-edge. Note that each union can lead to multiple hyper-edges. Hyper-edges in which a participation threshold (see section 2) was met were considered to be a shared hyper-edge or else was considered as a condition specific to the condition, which contained the largest proportion of raw edges in the hyperedge. (c) The proportion of shared and feature-specific edges for each frequency band. (d) Shared hyperedge networks for the θ and α-band frequency bands, visualized on the flattened cortical surface.

In addition to being feature-selective (Fig. 2c), we hypothesized that synchronization networks could also play a general content-agnostic role in VWM. We hypothesized that θ and α-band network synchronization could reflect the top-down selection of the memorized contents given their role in top-down control (D’Andrea et al., 2019; Lobier et al., 2018; Sadaghiani et al., 2019). Hence, we posited that the top-down executive network should be shared, i.e. connections should transcend across different memorized contents while the representational contents of VWM should be specific to the memorized feature information. We thus developed a novel graph-theory based approach to identify subnetworks that were shared across all VWM conditions and that were content-specific (Fig. 4a–b). This approach was implemented on synchronization networks averaged across load conditions. More specifically, we used hyperedge bundling (S. H. Wang et al., 2018) on an interim union matrix that combined edges from all feature conditions averaged across loads to extract the subgraphs that were specific to each memorized feature (Fig. 4a) and subgraphs that were shared across all three features (Fig. 4b).

Importantly, we found both shared and feature-specific subgraphs for both θ and α-band networks (Fig. 4c). This result indicated that within a frequency band, distinct sub-networks (subgraphs) can mediate distinct functions of VWM. While α-band networks consisted of feature-specific subgraphs especially for shape, θ-band networks consisted of feature-specific subgraphs especially for location. These results underscore frequency-specific network synchronization in maintaining specific VWM contents. Crucially, for both θ and α-band networks, the majority of edges were shared across the features, that is, content-agnostic. Importantly, these shared subnetworks coupled nodes in the attentional and fronto-parietal networks (Fig. 4d). For the shared θ-band desynchronization network, the most central hubs were the left and right occipitotemporal gyrus (OTG), which connected to the postcentral sulcus (poCS) of DAN and frontomarginal gyrus (mrgF) of FPN. For the shared α-band synchronization network, the most central hubs were the right FEF and left intrapariental sulcus (intPS), which are both key nodes of the DAN and were also connected to each other. Supplementary Table S1 lists the most central parcels of the shared networks, along with the functional subsystem of each parcel. The presence of both shared and content specific subgraphs within the α-band synchronization and θ-band desynchronization networks suggest that these networks enable the top-down selection of remembered contents.

### 3.5 α-band network synchronization predicts individual behavioral performance

**Fig. 5.**
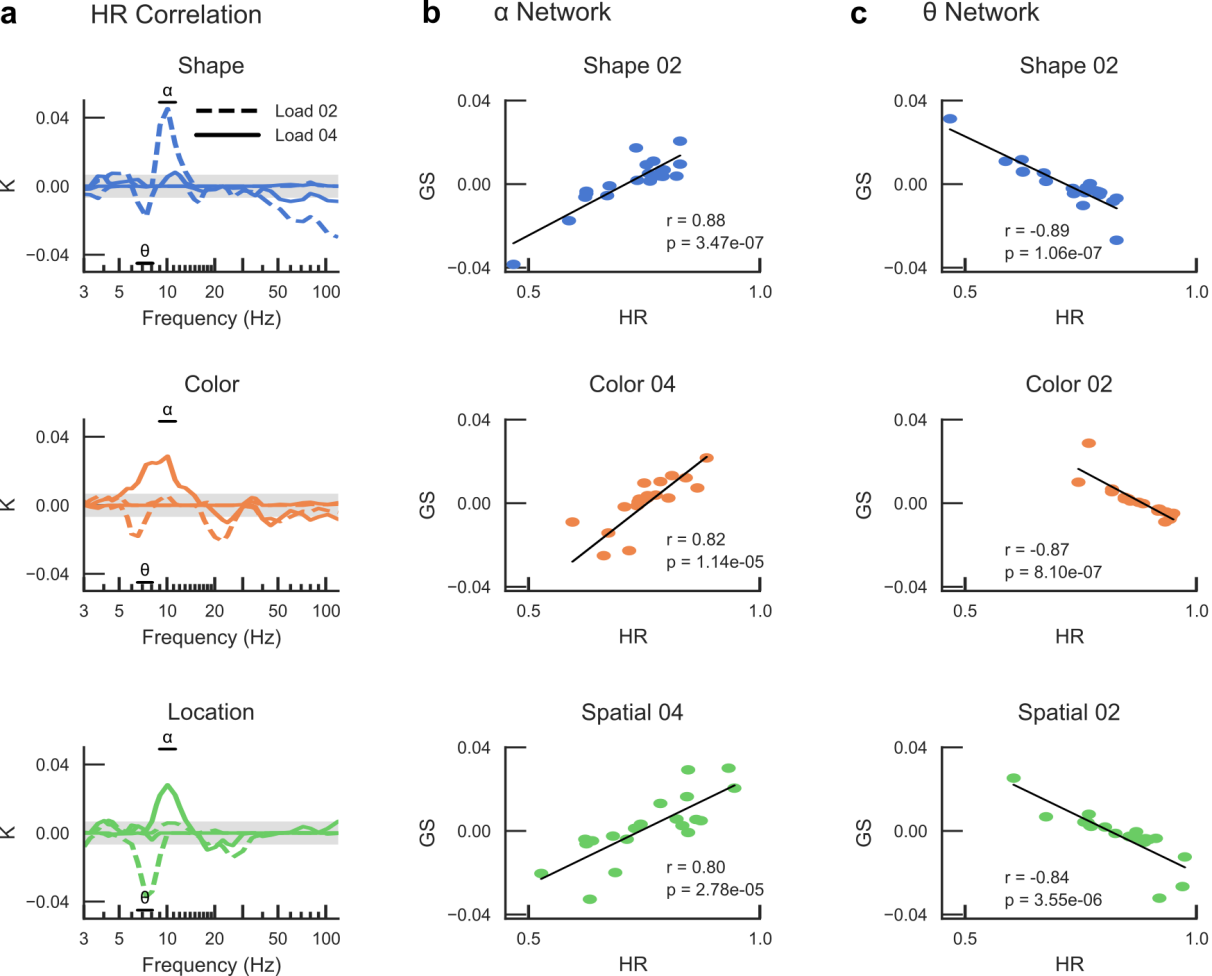
α and θ-band synchronization networks are correlated with individual behavioral performance. (a) Connection density for the significant correlations between the strengths of synchronization and Hit Rates (HR) (Pearson correlation test, *p* < 0.05). Edge iPLV values were correlated positively with HR in the α-band and negatively correlated in the θ-band for all three features. (b) The individual graph strengths (*GS*), that is, the summed strength of significant edges, as a function of the individual HRs. In the α-band (9–11 Hz), there was a strong positive correlation for all conditions. (c) Same as (b) but graph strength was extracted from the significantly suppressed edges in the θ-band (6.5–8 Hz). A strong negative correlation was observed between the graph strength in the θ-band and individual HR.

To address the behavioral relevance of the modulation in α-band synchronization and in θ-band desynchronization, we computed the correlation between each individual subject’s behavioral performance as indexed by the Hit Rate (HR), and the strength of inter-areal synchronization. We computed edge density, *K*, as the proportion of edges that were significantly correlated with HR. For all features, we observed a positive correlation between individual behavioral performance and inter-areal synchronization in the α-band and a negative correlation in the θ-band, as expected (Pearson correlation test, *p* < 0.05) (Fig. 5a). Post-hoc correlations between HR and the *GS* of all significant edges in the α-band and θ-bands, as shown in Figure 5a, were then performed. Significant correlations were observed in the α-band for load 4 of Color (*r* = 0.82, *p* = 1.14e-05) and Location (*r* = 0.80, *p* = 2.78e-05) and load 2 of Shape (*r* = 0.88, *p* = 3.47e-07) with matching difficulty (Fig. 5b) and in the θ-band for load 2 of Shape (*r* = -0.89, *p* = 1.06e-07), Color (*r* = -0.87, *p* = 8.10e-07) and Location (*r* = -0.84, *p* = 3.55e-06) (Fig. 5c). This indicated that the α-band synchronization GS and θ-band desynchronization GS at the individual level were correlated with individual HR, demonstrating the strong behavioral relevance of both α-band synchronization and θ-desynchronization, as would be expected if these networks are central for selecting the memorized VWM contents. In contrast, there was only a minor correlation of α-band oscillation amplitudes with HR, but a stronger negative correlation of β- and γ-band amplitudes with HR (Supplementary Fig. S10). This highlights the role of higher frequency oscillations at the local level in the maintenance of visual information in VWM compared to the network synchronization in the lower frequencies.

### 3.6 The memorized visual feature can be decoded from α-band synchronization patterns

**Fig. 6.**
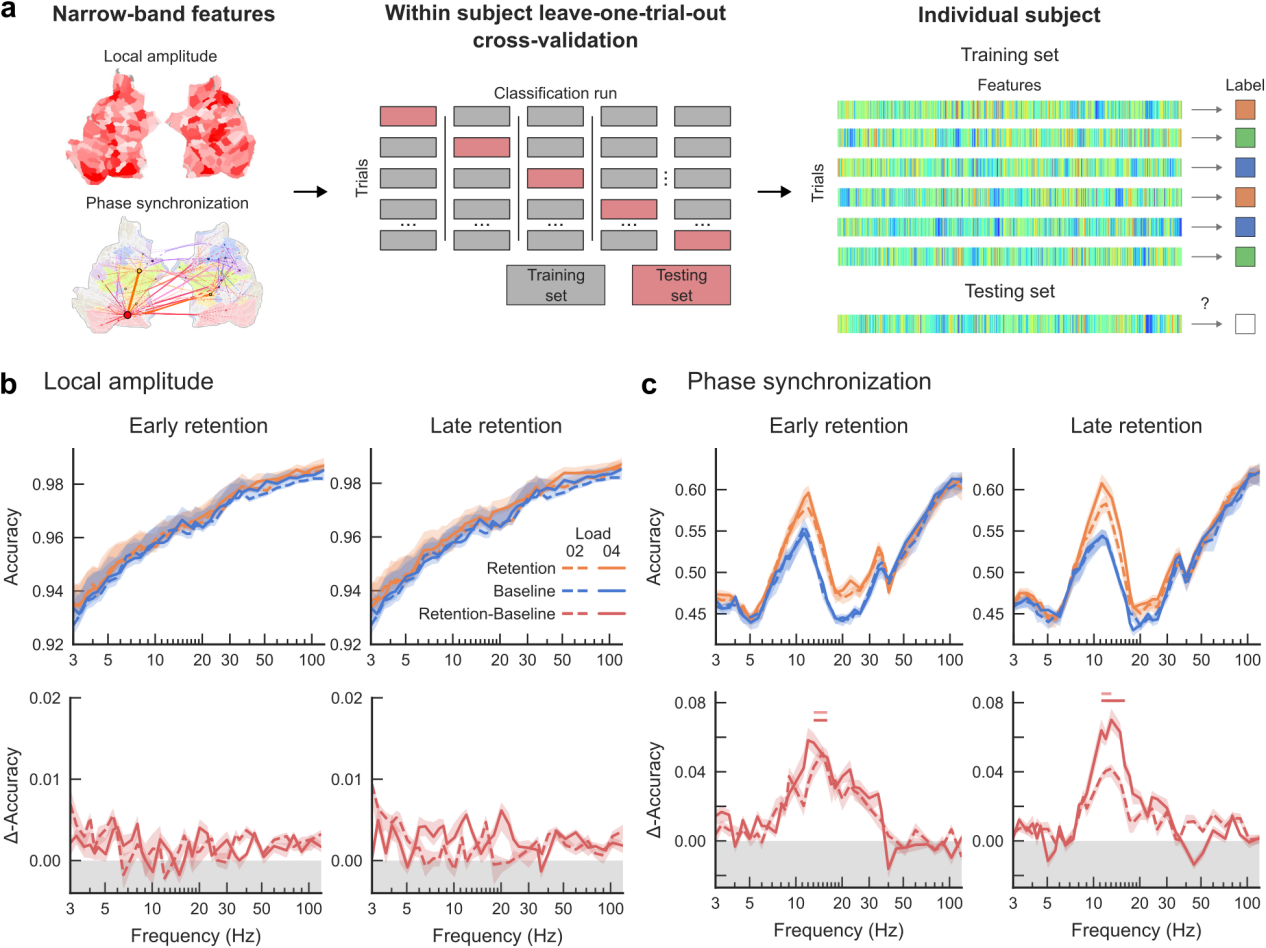
The memorized visual feature can be decoded from α and β-band synchronization patterns. (a) Local amplitude and inter-areal phase synchronization were used as features for multi-label supervised learning to classify trials of each condition (Shape, Color, and Location) within subjects. A random forest classifier was trained on local amplitudes and phase synchronization of each trial for 38 frequency bands within each subject and tested using leave-one-trial-out cross validation. (b) Classification of local amplitudes was performed separately for loads 2 and 4 (dashed and solid lines, resp.) and for retention (orange lines) and baseline (blue lines) time-windows. Difference curves (lower row, red lines) indicate differences in decoding accuracy between retention and baseline windows. Accuracy is the proportion of trials correctly classified from the total number of tested trials. Horizontal bars (light red: load 2, dark red: load 4) indicate significant differences between accuracy of retention and baseline (Two-tailed Wilcoxon signed-rank test, *p* < 0.05). (c) Same as (b) but classification was performed for inter-areal synchronization of each trial.

The previous analysis had established that α-band synchronization was feature-selective. To obtain validation on whether synchronization would contain information unique to the memorized feature that would enable decoding, we used machine learning (ML) analysis. A multivariate random forest classifier was trained on synchronization patterns on every trial for each frequency band and for each subject. Significance was then tested using the leave-one-out cross validation (LOOCV) method (Fig. 6a). Classification accuracy of the memorized feature in both retention windows and for both load conditions was highest in the α-band (Fig. 6c, top). However, the classification accuracy was above chance-level both for the baseline and retention period time windows. This likely reflects the block effect whereby the participants memorized different visual features in separate experimental blocks. To disentangle decoding performance due to global task effects, we estimated the difference in the classification accuracies between retention and baseline time-windows (Fig. 6c, bottom). This revealed significantly greater accuracies for the retention period between 10–30 Hz (two-tailed Wilcoxon signed-rank test, *p* < 0.05), with the greatest improvement in classification accuracy in the α-band.

The classifier analysis was also applied to amplitudes of each trial. Classification accuracies in both baseline and retention time-windows converged toward 0.98 (Fig. 6b, top), but the classification accuracy between retention and baseline data did not differ (Fig. 6b, bottom) indicating that this cannot be attributable to information in the retention period. While these data overall demonstrate that network synchronization is a reliable measure of VWM contents unlike local oscillations amplitudes, it should be noted that the single-trial feature vectors used for synchronization (1 x 1000 vectors) were larger than used for oscillation amplitudes (1 x 400 vectors) which makes the direct comparison difficult.

## 4 Discussion

Long-range phase synchronization across WM-related brain areas has been established to be fundamental for VWM, as demonstrated by EEG/MEG source connectivity analysis (Ericson et al., 2024; Mamashli et al., 2021; J. M. Palva et al., 2010; Sato et al., 2018; Sattelberger et al., 2024) and local field potential (LFP) recordings in monkeys (Liebe et al., 2012; Salazar et al., 2012). However, whether oscillatory networks would track the contents of VWM or reflect attention and executive control functions has remained unknown. Using a parametric VWM task controlling for the memorized features, combined with advanced network analysis approaches, we found that α-band synchronization networks track VWM contents. More precisely, we identified content-specific subnetworks of α-band synchronization and subnetworks that were shared across feature-conditions. This finding shows that α-band network synchronization reflects the representational functions of VWM (Baddeley, 2012; D’Esposito & Postle, 2015; Klingberg, 2010).

This result was surprising and not aligned with the traditional functional significance of local oscillations given that local β- and γ-band frequencies have been related to the representation of sensory information (Fries et al., 2002; Muthukumaraswamy & Singh, 2013) and memory contents (Honkanen et al., 2015; Morgan et al., 2011), whereas both local α-band oscillations (Capilla et al., 2014; Gould et al., 2011; Thut et al., 2006) and inter-areal α-band synchronization (Gregoriou et al., 2009; Lobier et al., 2018) are generally associated with attention, especially in the spatial domain. However, this study demonstrates that, similar to findings in auditory (Ahveninen et al., 2023; Mamashli et al., 2021) and verbal (Rossi et al., 2023) WM and that of LFP in monkeys (Salazar et al., 2012), long-range synchronization tracks the content of human VWM. Crucially, we demonstrate that α-band network synchronization, which was sensitive to and tracked VWM contents, was localized to subnetworks (subgraphs) in functional visual areas responsible for the processing for their respective features (Riesenhuber & Poggio, 2000, 2002) although connections were also found involving visual areas processing the distracting visual features. In addition, also connections from these functional visual areas with the frontoparietal network tracked VWM contents. These findings are in line with earlier work showing that VWM contents can be tracked in both the sensory cortices and in the PFC (Christophel et al., 2017; Serences, 2016). Our findings were further validated by ML analysis, which revealed that feature-specific synchronization enables decoding of memory content. However, the ML analysis also decoded contents during the baseline period, likely due to the block design, whereby the participant had an active memory representation across the whole block. In contrast to previous studies (Chen et al., 2022; Elshafei et al., 2022), we found no decoding for local oscillation amplitudes.

Our results can be explained by a framework in which α-band network synchronization implements top-down control to select for the features to be remembered. In line with this hypothesis, α-band synchronization subgraphs in the fronto-parietal network were shared across conditions in which different contents were memorized. This suggests that α-band network synchronization forms the executive core of VWM. The main hubs of the shared network, transcending across different conditions, were found in the frontoparietal control systems (Gratton et al., 2018; Power & Petersen, 2013) with the main network hubs in the IPS and FEF of the DAN (Corbetta & Shulman, 2002). Due to its multiplexing role, α-band network synchronization could implement the top-down selection of the memorized contents. This hypothesis is in line with the role of α-synchronization in attentional top-down control (D’Andrea et al., 2019; Lobier et al., 2018; Mishra et al., 2021; Sadaghiani et al., 2019) and the role of frontoparietal regions in controlling the prioritization of information in visual WM (Sahan et al., 2019) and in implementing spatial computing for the control of WM (Lundqvist et al., 2023). Furthermore, this framework aligns with the content-independent pointer system of WM that supports the attentive tracking of objects but not the content of the objects (Thyer et al., 2022). The presence of a shared executive network across different VWM contents is also analogous to the supramodal shared network across different modalities for conscious access and across tasks (Deco et al., 2021; Sanchez et al., 2020), which suggest a similar organizational principle for WM and perception. We advance here that the shared connections of α-band synchronization reflect the top-down executive network that enables the selection of the to-be-remembered visual contents, as reflected in content-specific synchronization in the visual cortices. The functional significance of α-band network synchronization in top-down control differs from the general view that alpha-band oscillations and synchronization would mainly have an inhibitory role, to protect memorized information from external interferences (Jensen, 2024; Jensen & Mazaheri, 2010). There are two explanations for these differences. First, α-band oscillation amplitudes and network synchronization may carry out distinct roles in the inhibition vs. top-down control of information (S. Palva & Palva, 2007, 2011). Second, canonical oscillatory frequencies may have multiple sources with differential functional roles, as has been established for visual attention (Benwell et al., 2017; Cruz et al., 2025; Iemi et al., 2017; Trajkovic et al., 2024; van Ede et al., 2017).

Both α-band synchronization and θ-band desynchronization were correlated with individual behavioral performance. High performers exhibited greater α-band synchronization and θ-band desynchronization than low performers, demonstrating the functional significance of inter-areal network synchronization in the maintenance of content-specific feature representations. α-band synchronization was strong during the late retention period which may reflect the refreshing of VWM contents, a post-consolidation process during maintenance that strengthens the memoranda and prevents loss of information (Morey & Cowan, 2018).

In contrast to many previous studies which have found θ-band synchronization during VWM, particularly in the hippocampus (Heusser et al., 2016), but also in cortex (Bahramisharif et al., 2018; Berger et al., 2019; Sarnthein et al., 1998), we found θ-band desynchronization. This finding is unlikely to be explained by a smaller signal-to-noise ratio, as θ-band and α-band oscillation amplitudes were similar. The lack of θ-band synchronization is similar to that found in our previous studies using delayed match-to-sample VWM tasks (J. M. Palva et al., 2010; Sattelberger et al., 2024). In particular, θ-band oscillations are thought to reflect control mechanisms in WM (Berger & Sauseng, 2022; Sauseng et al., 2010), which the delayed match-to-sample VWM task used in the present study did not involve.

The combination of α-band synchronization and θ-band desynchronization in the present task may be complementary. This could reflect the opposing demands of VWM in maintaining the internal WM representations and in inhibiting the external sensory stimulation (Van Ede & Nobre, 2023). This could be achieved via concurrent α-band synchronization, and θ-band desynchronization in the visual areas suppressing incoming new sensory information (Johnson et al., 2023) similarly to that have been proposed previously to α-band oscillation amplitudes (Jensen, 2024; Jensen & Mazaheri, 2010). Differences in canonical frequency bands in this respect may arise due to small spectral differences caused by task differences as well as by different analytic pipelines. Thus, α-band synchronization and θ-band desynchronization may operate in a complementary fashion to maintain representations and simultaneously prevent interference from incoming sensory information.

Overall, our results establish that large-scale α-band synchronization reflects not only the “What” (*i.e.*, the contents) of visual working memory, but also suggests the existence of a shared control network that transcends specific memory contents. We propose a model where α-band synchronization implements top-down selection of memorized visual information based on behavioral needs. The concept of a network that is not a silo for a particular type of information or content would explain information leaks between memories across different types of content (Robertson, 2022) as well as between different cognitive tasks (Ericson et al., 2024). Thus, long-range synchronization may serve multiplexed roles and reflect both working memory contents and executive demands, which together provide the key architectural features of visual working memory.

## Supplementary Material

Supplementary Material

## Data Availability

Raw electrophysiological data cannot be shared publicly due to regulations imposed by the Ethical permission but can be shared for collaborative efforts upon request. The single-subject connectomes underlying the results, along with the plotted values in these figures and supporting files used in the processing of MEG data, is publicly available at a DataDryad repository (https://doi.org/10.5061/dryad.np5hqc00n). All code used in this work to produce results, run the statistical analyses, and create the final figures can be found at https://github.com/palvalab/vwm_synchronization. All data are available in the main text or the Supplementary Materials.
