# Supplementary material for: Top-down selection of visual working memory contents is supported by alpha-band phase-synchronized oscillatory networks
Source: Imaging Neurosci (Camb). 2025 Dec 23;3:IMAG.a.1034. doi: 10.1162/IMAG.a.1034 (PMC12723408; doi:10.1162/IMAG.a.1034)
Supplement: Supplementary Material [file IMAG.a.1034_supp.pdf]

Supplementary Materials

Top-down selection of visual working memory contents is supported by alpha-band phase-synchronized oscillatory networks

Hamed Haque, Sheng H Wang, Felix Siebenhühner, Edwin M. Robertson, J. Matias Palva, & Satu Palva

This PDF file includes:

Figures S1 to S10
Table S1

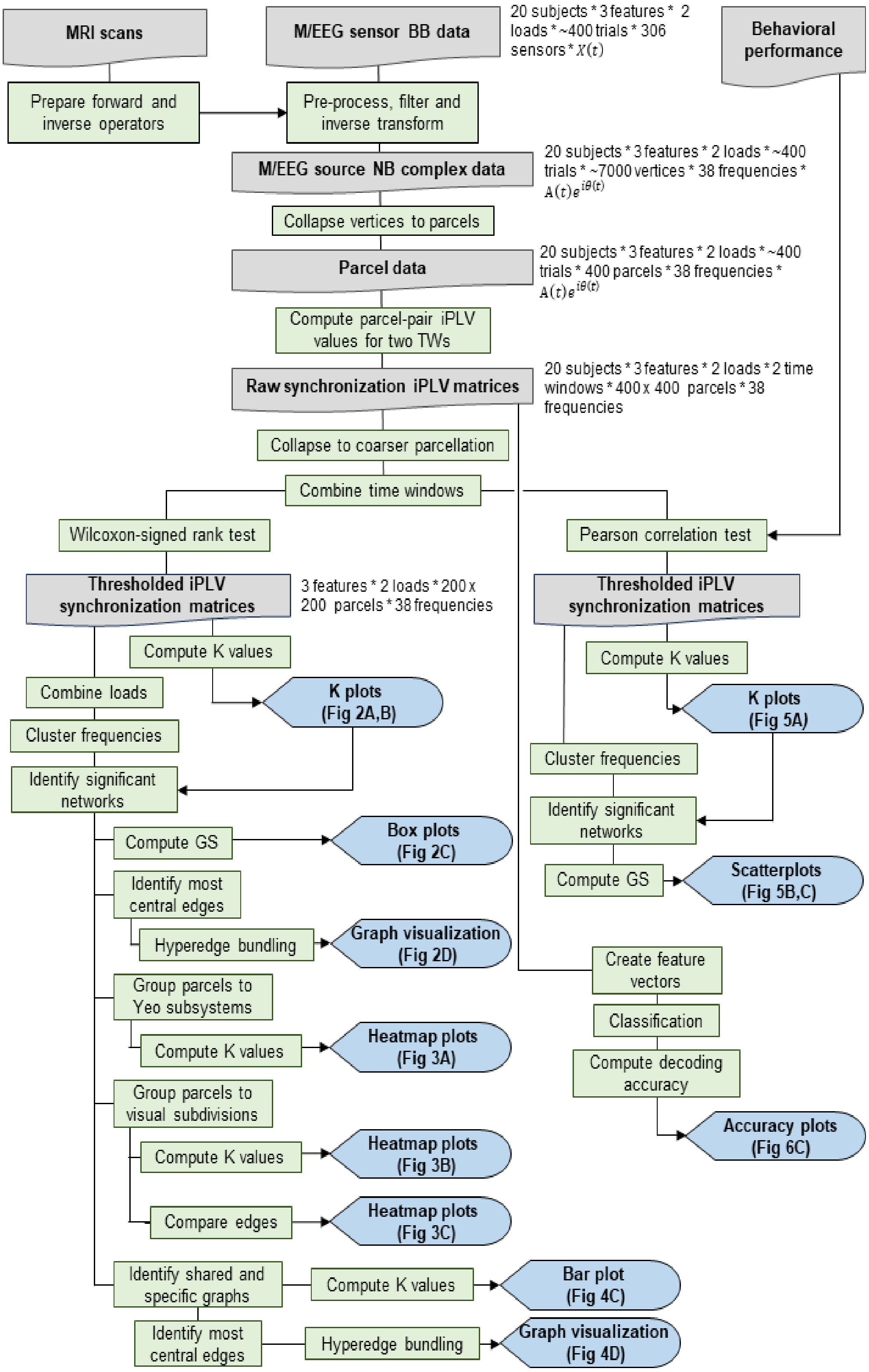

Fig. S1. Analysis pipeline flowchart. A schematic overview of the analysis steps underlying the phase synchronization results shown in Figs. 2-6. Gray boxes refer to data, green to processes, and blue to figures.

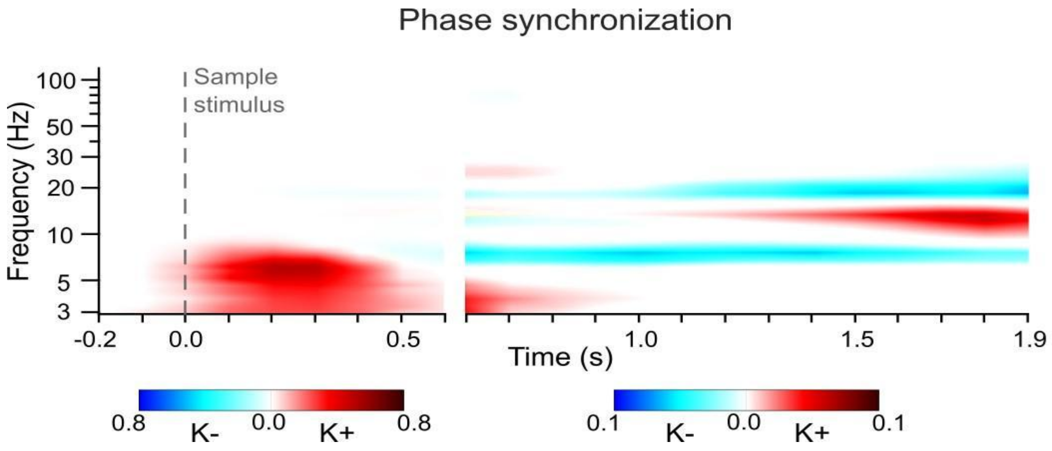

Fig. S2. Grand averaged synchronization during VWM retention. Time-frequency representation (TFR) of significant differences in inter-areal synchronization compared to the prestimulus baseline (Two-tailed Wilcoxon signed rank test, $p < 0.05$). Trials from all three visual features (Shape, Color, and Location) and load conditions (2 and 4 objects) were included.

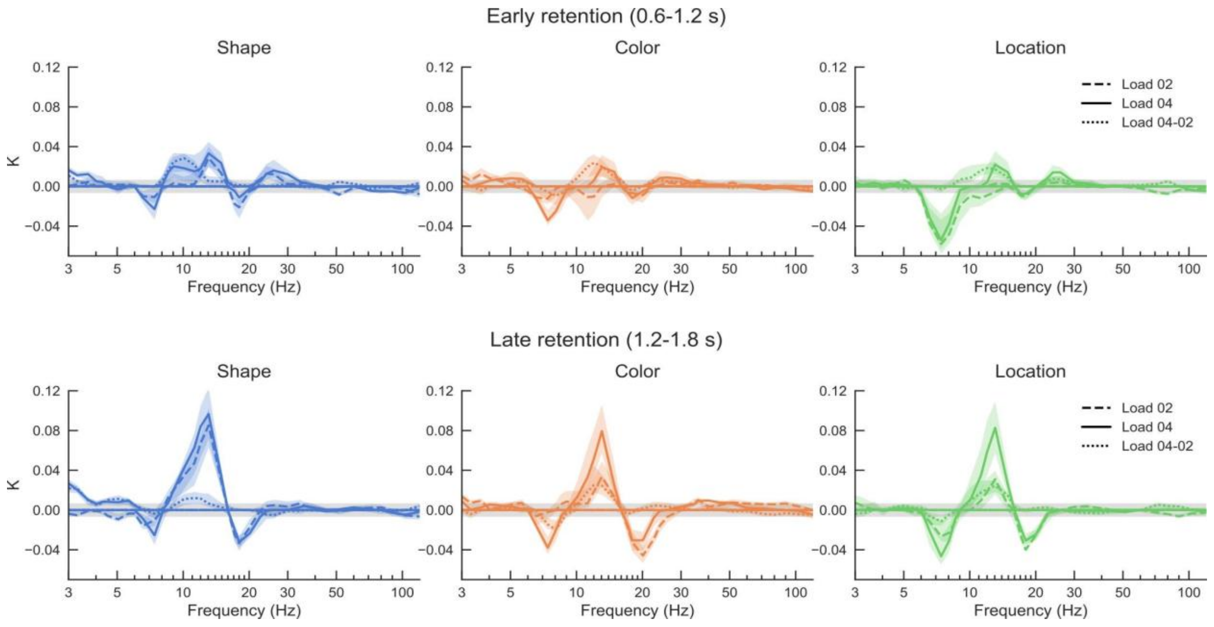

Fig. S3. Inter-areal synchronization in early and late retention periods. Connection density (*K*) compared to baseline separately for early and late retention periods (Two-tailed Wilcoxon signed rank test, $p < 0.05$). Positive values show connections that are increased and negative values, connections that are decreased compared to baseline. Shaded gray area indicates values below the Q-level of 0.00672.

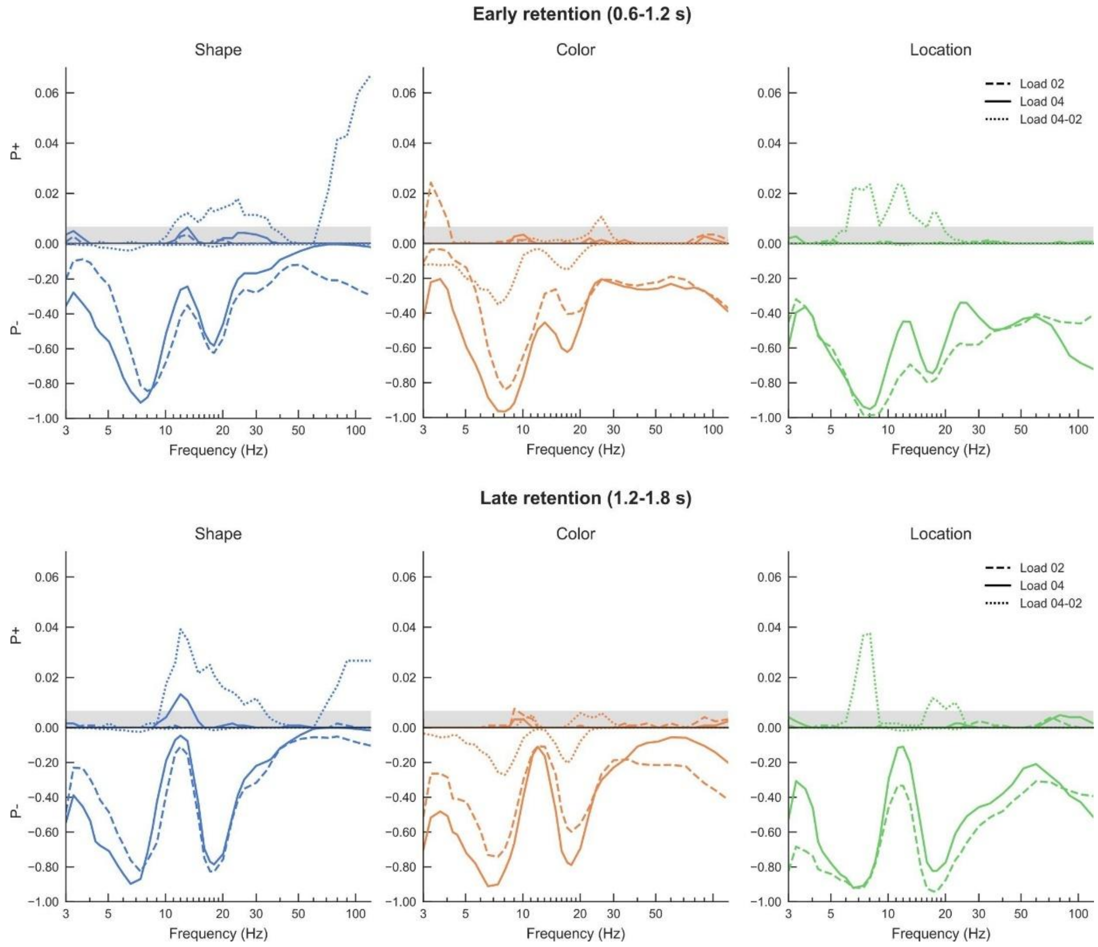

Fig. S4. Oscillation amplitudes in early and late VWM retention. The fraction of cortical parcels in which local oscillation amplitudes during the retention period were significantly greater (P+) or lower (P-) than during the baseline period (Wilcoxon two-tailed signed-rank test, $p < 0.05$). Oscillation amplitudes of each frequency were estimated during the Early and Late retention windows. Shaded gray area indicates values below the Q-level of 0.00672. Negative values indicate significant edges at the negative tail (P-).

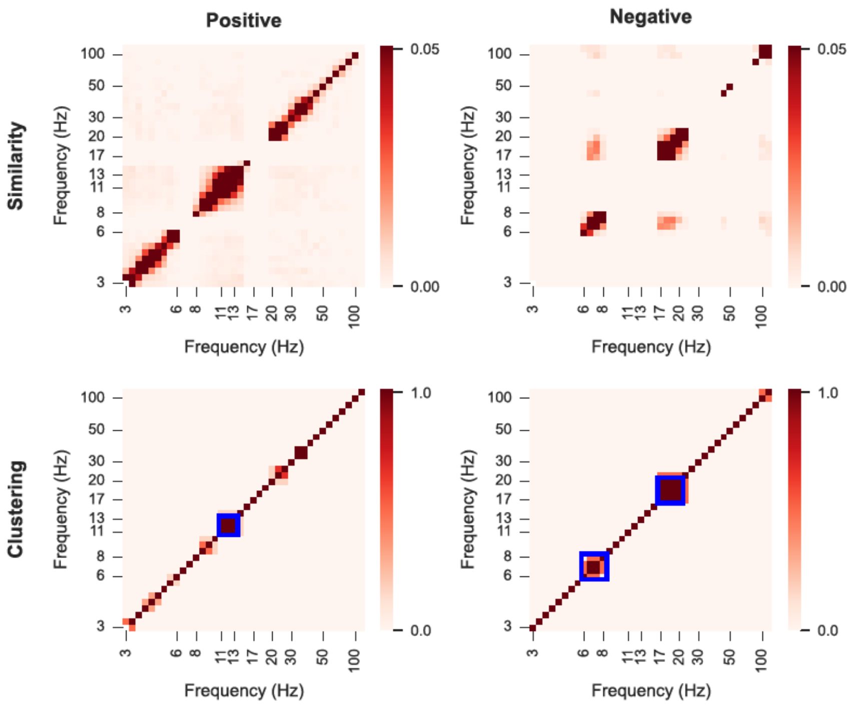

Fig. S5. Edge similarity estimated with hierarchical clustering across frequencies. Edge similarity was used to group in adjacent frequencies into frequency bands based on spatial similarity of the adjacent matrices using hierarchical clustering. The blue boxes indicate clustering of frequencies into the high-α- (11-13 Hz), θ- (6-8 Hz), and β-bands (17-20 Hz).

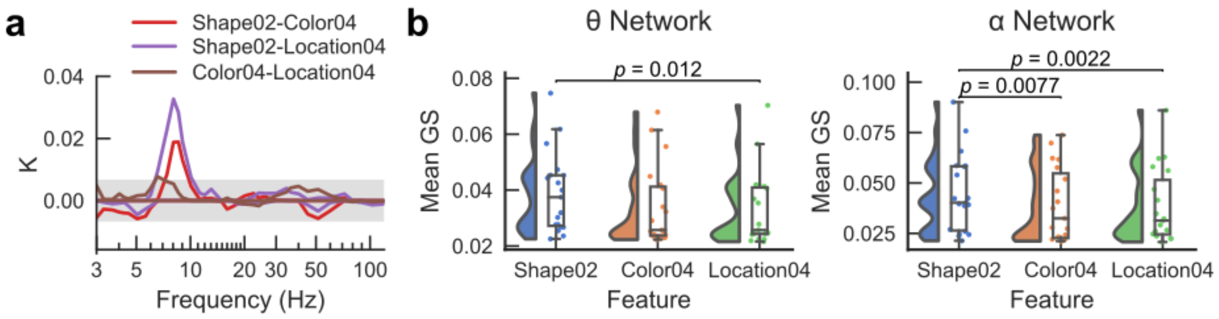

Fig. S6. Differences in graph strength between visual features do not reflect task difficulty. **a** Edge density for the significant differences between the inter-areal networks of Shape02 (object load of 2 items), Color04, and Location04. **b** Distribution of individual graph strength (GS) values for the θ- and α-band networks for the Shape02, Color04, and Location04, with the edges selected based on (a). Two-tailed repeated measures t-test was performed between each pair of features.

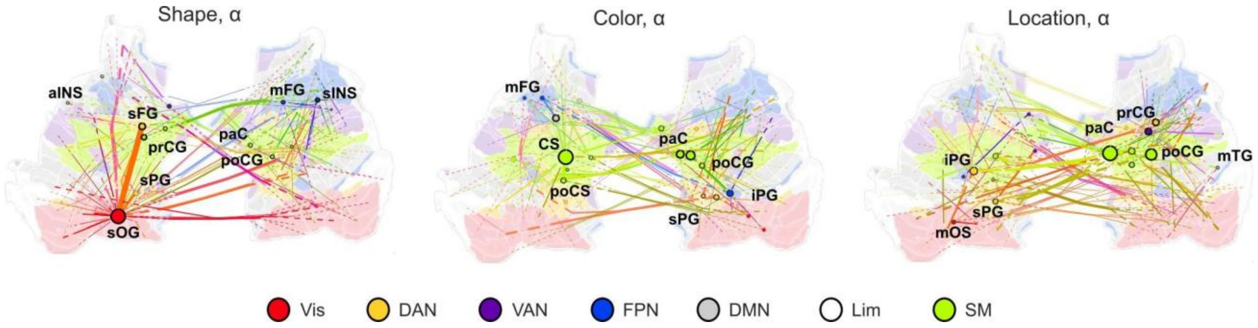

Fig. S7. α-band synchronization based on full graphs. Significant connections of α-band synchronization for the full (complete) graph. Figures display the 200 edges with the highest Edge Betweenness Centrality, bundled as described in Methods. Node size represents node Betweenness Centrality while the color of nodes, edges, and cortical surfaces represent different functional systems as indicated below. DAN = dorsal attention network, VAN = ventral attention network, FPN = frontoparietal network. For clarity, node names were restricted to nodes with the 20% highest node degree.

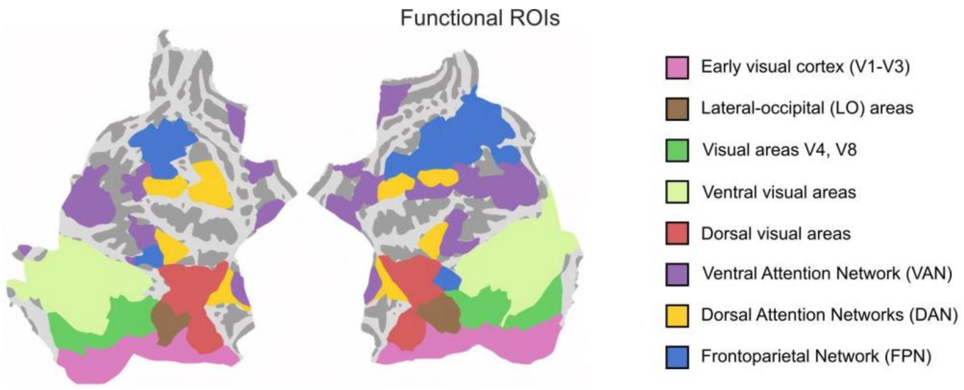

Fig. S8. Cortical parcels within functional systems. Parcels of the Destrieux atlas defined in the Colors define parcels that are within V1-V3, lateral-occipital (LO) areas, V4, V8, ventral visual areas, dorsal visual areas, ventral attention network (VAN), dorsal attention network (DAN), and frontoparietal network (FPN) as used in Fig. 2.

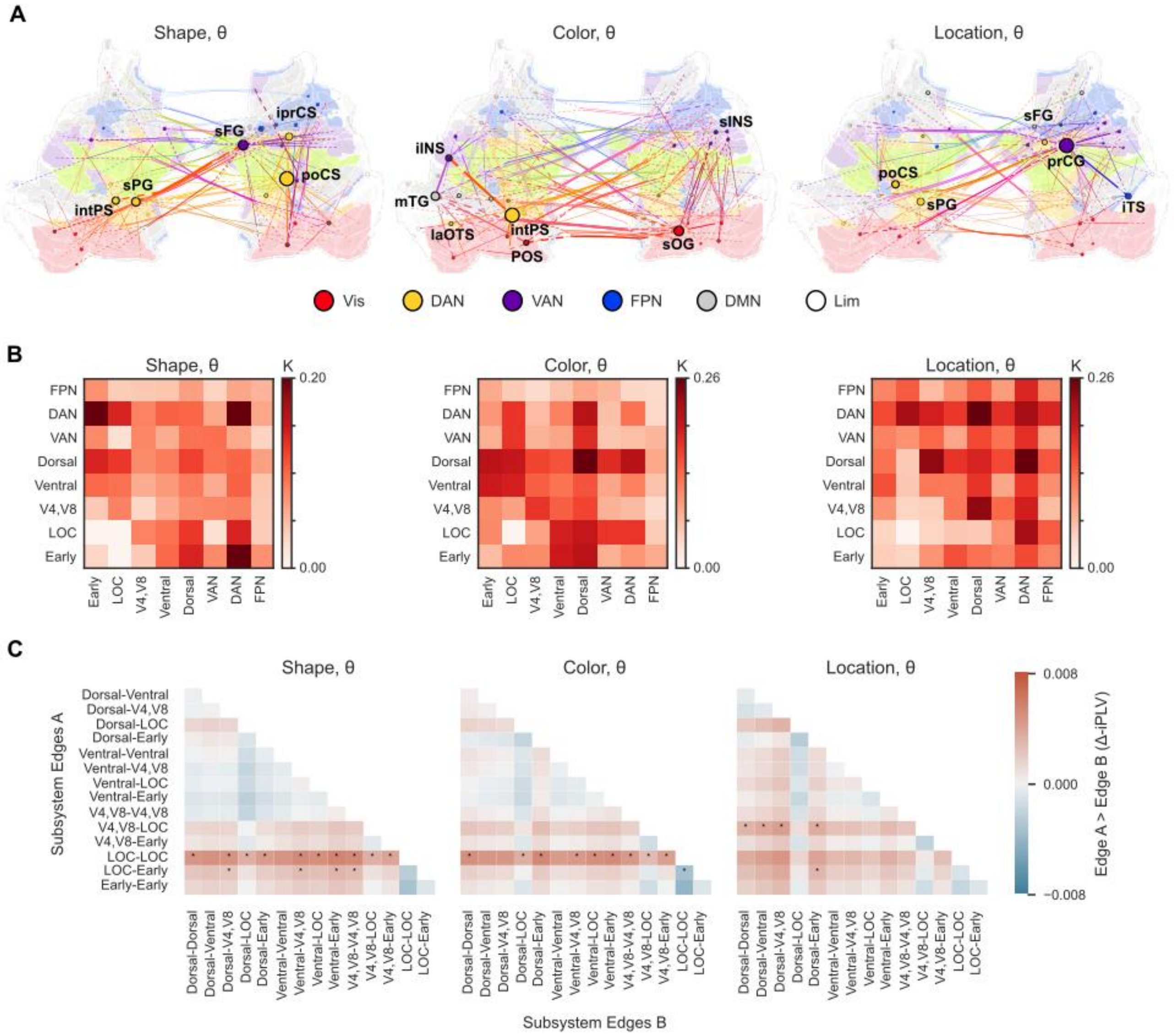

Fig. S9. θ-band desynchronization connected the fronto-parietal attention networks to distinct visual functional networks. (**A**) Significant connections of θ-band desynchronization for each visual feature displayed on a flattened cortical surface. (**B**) Connection density for the retention period of single conditions (Shape, Color, Location) for each within and between functional networks. (**C**) The differences in mean phase desynchronization (Δ-iPLV) between visual subsystem edges (Edge A > Edge B). Red color indicates stronger desynchronization for edges in subsystem A and blue for edges in subsystem B. θ-band desynchronization was consistently stronger for Shape and Color within the LOC.

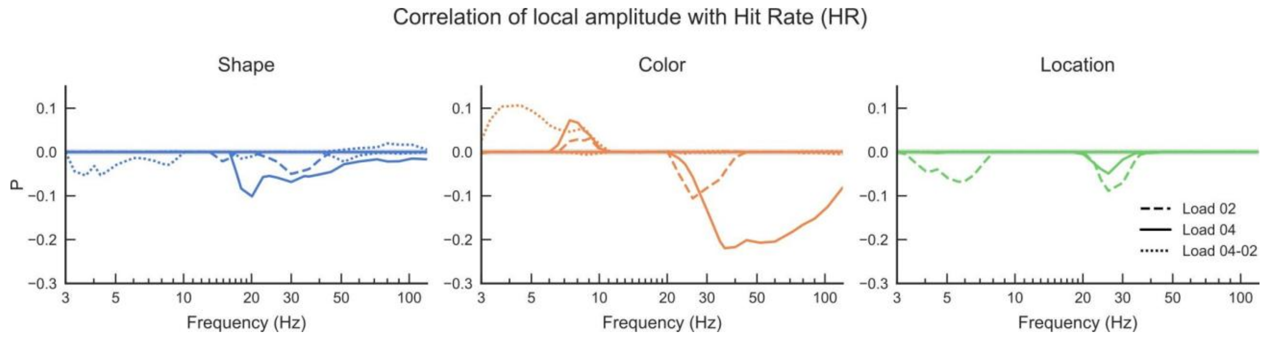

Fig. S10. Correlation of oscillation amplitudes with individual Hit Rate (HR). The fraction of cortical parcels in which local oscillation amplitudes during the retention period were significantly correlated with individual HR (Spearman rank correlation test, $p > 0.05$). β-band amplitudes in all three features were negatively correlated with HR and γ-band amplitudes in Shape and Color were negatively correlated with HR.

Network	Parcel name	Label	Hemifield	Degree	Subsystem
Shape, α	Superior occipital gyrus	sOG	Left	56	Visual
	Frontal eye fields	FEF	Left	24	DAN
	Middle-anterior cingulate	aCIm	Left	10	VAN
	Intraparietal sulcus	intPS	Right	10	DAN
	Middle frontal gyrus	mFG	Right	9	FPN
	Inferior frontal gyrus	iFG	Right	8	FPN
	Superior parietal gyrus	sPG	Left	7	DAN
Color, α	Angular gyrus	iPGang	Right	30	FPN
	Intraparietal sulcus	intPS	Right	24	DAN
	Middle frontal gyrus	mFG	Left	18	DMN
	Frontal eye fields	FEF	Right	16	DAN
	Frontal eye fields	FEF	Left	11	DAN
	Frontomarginal gyrus	mrgF	Right	9	FPN
	Orbital sulcus	orbS	Left	9	FPN
Location, α	Precentral gyrus	prCG	Right	29	VAN
	Precentral sulcus	iprCS	Right	19	DAN
	Supramarginal gyrus	iPGsup	Left	17	FPN
	Marginal cingulate sulcus	CISmrg	Left	15	VAN
	Postcentral sulcus	poCS	Left	15	DAN
	Frontal eye fields	FEF	Right	12	DAN
	Superior parietal gyrus	sPG	Left	11	DAN
Shared, α	Frontal eye fields	FEF	Right	32	DAN
	Intraparietal sulcus	intPS	Left	21	DAN
	Supramarginal gyrus	iPGsup	Right	20	VAN
	Inferior frontal gyrus	iFG	Left	16	DMN
	Angular gyrus	iPGang	Right	16	FPN
	Precentral gyrus	prCG	Right	15	VAN
	Occipitotemporal gyrus	OTG	Left	11	Limbic
Shared, θ	Occipitotemporal gyrus	OTG	Left	19	Limbic
	Occipitotemporal gyrus	OTG	Right	17	Limbic
	Middle-anterior cingulate	aCIm	Right	14	VAN
	Postcentral sulcus	poCS	Left	12	DAN
	Inferior occipital gyrus and sulcus	iO	Left	12	Visual
	Frontomarginal gyrus	mrgF	Right	10	FPN
	Superior temporal sulcus	sTS	Left	10	DMN

Table S1. List of the most central nodes for each of the inter-areal networks. For each of the networks visualized in Fig. 2D and 4D, the parcels with the highest degree are listed. The 'Parcel name' states the full name of the parcel and 'Label' corresponds to the abbreviation used in network visualization. The subsystem in which the parcel belongs to is provided using the following abbreviations: visual network (Visual); dorsal attention network (DAN); ventral attention network (VAN); limbic network (Lim); fronto-parietal network (FPN); default mode network (DMN).
